# Tuberculosis Meningitis in People Living with HIV/AIDS in a Health Center in the Brazilian Amazon: A Silent Disease

**DOI:** 10.1155/2022/8048310

**Published:** 2022-03-14

**Authors:** José Eudes de Carvalho Neri, Aline Cristine Passos de Souza, Ana Carolina Paes Boulhosa, Rafaela Caroline Amador Ferreira, Chester Darlan de Souza Soares, Cleuson Vieira Costa, Julius Caesar Mendes Soares Monteiro

**Affiliations:** Federal University of Para, Belém, Brazil

## Abstract

**Introduction:**

Tuberculosis (TB) is one of the ten leading causes of death worldwide and the leading cause of infection in people living with the human immunodeficiency virus (HIV)/acquired immunodeficiency syndrome (AIDS) (PLWHA). It is a major public health problem in Brazil and worldwide.

**Methods:**

This was a case series study of five meningeal tuberculosis and PLWHA coinfection patients admitted between June 2019 and June 2020, in a public hospital in the northern region of Brazil. Associated with clinical cases, we propose a discussion of the different laboratory diagnostic methods available in Brazil, with the aim of increasing the diagnosis of this very serious disease, with high mortality.

**Results:**

The diagnosis of tuberculous meningitis is a challenge in clinical practice; thus, the clinical cases presented help the physician to recognize the signs and symptoms of the disease and improve the confirmatory diagnosis through acid-alcoholic resistant bacilli techniques, molecular testing, and mycobacteria culture in the cerebrospinal fluid.

**Conclusion:**

Knowing the diagnostic methods of tuberculous meningitis and its characteristics is of paramount importance to increase the correct diagnosis and reduce mortality in delayed treatment.

## 1. Introduction

Tuberculosis (TB) is one of the ten leading causes of death in the world, and the first cause of infection in people living with human immunodeficiency virus (HIV)/acquired immunodeficiency syndrome (AIDS) (PLWHA). It is a major public health problem in Brazil and worldwide. In 2019, 96005 new cases were reported nationwide, and 8774 of them had HIV coinfection [1].

The risk of TB increases 28 times in PLWHA, demonstrating the importance of being diagnosed with HIV infection regardless of age or site of infection. Tuberculous meningitis (TM) is the most lethal form of the disease, corresponding to 1% of all TB cases, with mortality close to 40% in PLWHA. The meningeal involvement results from the hematogenous dissemination of *Mycobacterium tuberculosis* from the primary pulmonary infection, with formation of small subpial and subependymal foci in the brain and spinal cord [2]. In some patients, these foci rupture and release bacteria into the subarachnoid space causing meningitis; the main complications are hydrocephalus and cerebral vasculitis. A delay in diagnosis of TM decreases the chances of cure, increasing the probability of sequelae. In 2018, the proportion of HIV testing in new TB cases in Brazil was 75%, with the highest proportion in the southern region (82.8%) [3].

## 2. Methodology

This was a case series study of five patients admitted between June 2019 and June 2020, at the Meningitis Diagnostic Unit of João de Barros Barreto Hospital, Belém, Pará, Brazil. Their diagnosis of HIV infection had been previously confirmed or was confirmed during hospitalization. All patients underwent lumbar puncture, biochemical, and cytological analyses of the cerebrospinal fluid (CSF), acid-alcoholic resistant bacilli (AARB) test, GeneXpert MTB/RIF (*Mycobacterium tuberculosis* and detect resistance to rifampicin) rapid molecular test for TB, UltraXpert MTB/RIF, and culture for mycobacteria. HIV infection was not confirmed by culture and RMT-TB in only one patient retreated for meningeal TB, since there was no access to his culture analysis from the first hospitalization. Data research was approved by the institution's ethics and research council (CAAE: 30566720.6.0000.0017).

## 3. Case Presentation

### 3.1. Case Report 1

A 29-year-old male patient was admitted in June 2019 due complaints of productive hemocytotic cough, daily afternoon fever, unintentional weight loss, hyporexia, asthenia for 3 months, progressive time-space disorientation, and lower limb paresthesia. He was diagnosed with HIV infection in the same month. He demonstrated CD4 count nadir 35 cells, CSF with positive AARB, positive RMT-TB, and positive culture for *M. tuberculosis*. Tuberculostatic treatment was initiated. Pulmonary involvement was confirmed with positive sputum smear tests for AARB and RMT-TB.

### 3.2. Case Report 2

**Figure 1:**
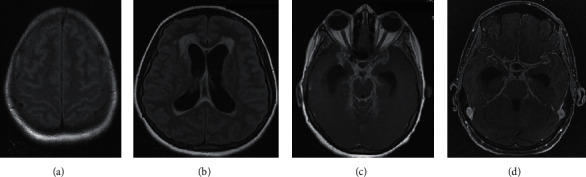
Nuclear magnetic resonance in case 2. (a) Hypersignal in the cerebral turns, bilaterally high convexity in T2 FLAIR. (b) Moderate dilated supratentorial ventricular system, associated with periventricular white matter hypersignal, probable CSF transudation on T2 FLAIR. (c)-(d) After administration of venous contrast, intense diffuse meningeal enhancement markedly in the regions of the cisterns of the base, axial T1, and Vol T1 3D, respectively.

A 32-year-old female patient, diagnosed with HIV/AIDS in 2017, was admitted in June 2018 because of headache, nausea, and disorientation progressing for 1 month; the patient had discontinued pulmonary TB treatment in the previous 3 months. She had a history of poor adherence to antiretroviral therapy, but with undetectable viral load and TCD4 lymphocyte count of <200 cells upon admission. Her CSF culture, AARB, and RMT-TB results were all positive. Specific therapy for TM with tuberculostatic drugs was initiated. She also demonstrated positive culture for mycobacteria, *M. tuberculosis* was detected, and sensitivity test showed resistance to isoniazid. Patient progressed with generalized tonic-clonic seizures and refractory hydrocephalus and was required to undergo a ventriculoperitoneal shunt in a reference service center. The patient's magnetic resonance (MR) images are shown in Figure 1, with the characteristic findings of TM described.

### 3.3. Case Report 3

A 29-year-old male patient, diagnosed with HIV/AIDS in 2018, was admitted in June 2019 due to intense headache, seizures, and lower limb paresis. The patient had a history of antiretroviral therapy abandonment and empirical TM treatment in 2018. On admission, the patient presented a viral load of >100,000 copies and a TCD4 lymphocyte count of <50 cells. CSF culture and AARB showed positive results. TM treatment was restarted with RHZE and corticotherapy. The patient progressed with significant motor improvement and seizure control. He underwent outpatient follow-up after hospital discharge.

### 3.4. Case Report 4

A 67-year-old male patient was diagnosed with HIV/AIDS in January 2020 and nadir CD4 32 cells, prompting the initiation of antiretroviral therapy. In April of the same year, he presented with weight loss, decreased level of consciousness, and afternoon fever for about 1 month. Clinical-radiological investigation with chest tomography suggested miliary TB, and MR imaging of the skull showed small multiple oval lesions without the mass effect. CSF culture, AARB, and RMT-TB showed positive results. Specific therapy was initiated, but the patient developed bronchoaspiration pneumonia and sepsis and eventually died during the same hospitalization.

### 3.5. Case Report 5

A 58-year-old male patient, diagnosed with HIV/AIDS in June 2020, during hospitalization, nadir CD4 26 cells, admitted with headache, fever for 1 month, and cervical lymphadenomegaly. CSF showed positive AARB and detectable RMT-TB. Specific plan was initiated with improvement of his initial conditions. He underwent outpatient follow-up after hospital discharge.

## 4. Discussion

Increased rate in TB/HIV coinfection tended to increase the number of TM cases in this population; therefore, it is extremely important to know the most reliable diagnostic methods available in the healthcare system. Bacteriological confirmation by the AARB, rapid molecular test, and culture are essential for the etiological definition of the case. Due to the severity of this disease, the beginning of treatment is often empirical, and the diagnostic investigation is not properly performed. On the contrary, late diagnosis leads to a considerable increase in lethality and sequelae [4].  Table 1 provides the clinical-epidemiological profile of the cases presented, with special attention to the low CD4 count cell in most cases, and the involvement of two or more sites. The cases diagnosed upon hospitalization could not be evaluated for antiretroviral therapy (ART) adherence before TM diagnosis.

The minimum amount of CSF to be collected for clinical analysis is 1 mL; however, the ideal volume is around 5–10 mL. A chemocytological pattern with inflammatory reaction is expected, with increased protein levels, hypoglycorrhachia, and increased number of lymphocytes, with a predominance of lymphomononuclear cells. The opening pressure is generally high, and the liquid has a turbid aspect [2, 4]. However, CSF study can be normal in up to 25% of the patients [5]. As given in Table 2, all patients presented increased cellularity, proteinorrhachia, and positive AARB test; the diagnoses of most of these patients were confirmed by the rapid molecular test or culture test. Only the diagnosis of case 3 was not confirmed, but the patient had already started empirical treatment in previous hospitalization.

**Figure 2:**
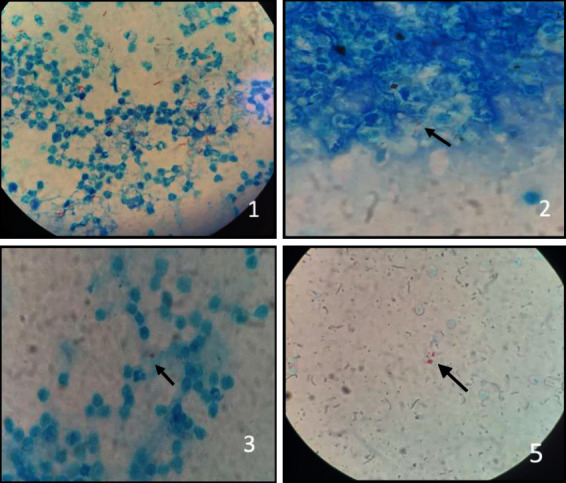
AFB staining slides, Ziehl–Neelsen technique, in CSF, optical microscope, 1000x. (a) *Mycobacterium tuberculosis*. (b) There was no growth. Arrows indicate the stained bacillus. Source: HUJBB laboratory data.

The first test discussed was the CSF *Mycobacterium* smear microscopy by AARB searching using the Ziehl–Neelsen technique. Developed over 100 years ago, it is still the most widely used method [6]. It has some advantages, such as low cost and possibility of be performed within 1 hour, but the sensitivity is about 10–20% in an examiner-dependent test. Some previous trials showed that the greater the sample volume, at least 6 mL, the greater the test sensitivity, reaching up to 60% [7, 8]. After the initiation of TM treatment, the bacilloscopy sensitivity is significantly reduced, usually after 5–15 days of therapy [9]. The five cases presented here had a positive AARB test at admission, and three of them had absence of AARB in the control CSF after treatment.  Figure 2 shows cases 1, 3, 4, and 5 AARB research glass slides under optical microscope on 1000× magnification.

CSF culture for *M. tuberculosis* is the gold standard for TM [10]. The test results are obtained within 3–5 weeks due to slow growth of the bacteria. The test sensitivity is around 50–70% [5]. PLWHA have higher culture positivity compared with HIV-uninfected patients, probably due to the higher bacilliferous load during TB/HIV coinfection [3]. The culture result is not typically used to determine whether to start treatment, but the test results are collected and analyzed to help confirm the diagnosis; the performance of the sensitivity test is also examined to identify for possible antimicrobial resistance [11].

Since 2014, the World Health Organization has strongly recommended the performance of the GeneXpert MTB/RIF molecular test for the diagnosis of meningeal TB [12]. The test had already been implemented since 2010 to examine the pulmonary samples from patients worldwide, including Brazil. It is conducted based on real-time polymerase chain reaction, which amplifies nucleic acids, and can detect 10 colony forming units (CFU)/mL. It has dual function, allows molecular detection of *M. tuberculosis* complex, and can identify resistance to rifampin, the main drug for TB treatment [9]. Initial studies showed 67% sensitivity in the general population and only 36% in PLWHA, demonstrating that the isolated use of this test did not exclude the disease, being necessary to associate other diagnostic methods [13]. Only one of the patients presented here showed no association between AARB research and detectable RMT-TB, but the patient had previously undergone irregular treatment. Recent meta-analysis identified 80.52% sensitivity and 97.8% specificity compared with the gold standard, without great differences among PLWHA [14]. It has some advantages, such as confirmation of the species, detection of rifampicin resistance, not examiner-dependent, fast result in about two and a half hours, and can be conducted in several health centers in Brazil [8, 9, 11].

In March 2017, the World Health Organization recommended the use of the new generation GeneXpert molecular test, UltraXpert MTB/RIF, which detects 100 CFU/mL of bacteria in the specimen, that is, 10× times higher than that using GeneXpert MTB/RIF [15]. A study conducted in Uganda with 129 HIV-infected patients and 23 patients with bacteriologically confirmed TM compared Xpert, UltraXpert, and culture and showed sensitivity rates of 45%, 95%, and 45%, respectively. This was significantly relevant and served as a basis for increasing the use of this method in the rapid and accurate diagnosis of TM [16]. Following the recommendation by the WHO on the use of the new generation GeneXpert molecular test, the Brazil National Health System also officially declared the use of UltraXpert as a replacement of Xpert in the diagnosis of pulmonary and extrapulmonary TB in her health units [11]. This declaration aimed at increasing the number of confirmed diagnostic TB cases to enable initiation of specific TB treatment as soon as possible [11].

## 5. Conclusion

TB is still one of the socially important neglected diseases, being associated with discrimination, delays in diagnosis, difficult access to treatments, and poor follow-up. When associated with HIV infection, it becomes an enormous challenge for the health system, not only nationally but also worldwide.

The main objective of this study was to ensure a correct and fast diagnosis to reduce the likelihood of sequelae. Among the small five case samples, one died during hospitalization, one was transferred for hydrocephalus correction, and three underwent outpatient follow-up with other comorbidities besides TM. The greatest challenge for the health team is to ensure daily adherence to the large amount of tuberculostatic and antiretroviral medications and prophylaxis for opportunistic infections, even after discharge.

The currently presented and previous used diagnostic methods for TB are already widely used in large health facilities in Brazil since the rate of TM countrywide cases in the general population is on the increase. The appropriate request and correct analysis of investigatory results for TB patients to enable reduction of empirical therapies and early detection of antimicrobial resistance and the mandatory screening of TB/HIV coinfection in all TM patients are strongly recommended.

## Figures and Tables

**Table 1:** Epidemiological profile of patients with tuberculous meningitis.

	Case 1	Case 2	Case 3	Case 4	Case 5
Sex	M	F	M	M	M
Age (years)	29	32	29	67	58
CD4 nadir	35 cells	315 cells	77 cells	32 cells	26 cells
HIV diagnosis time	To hospitalization	3 years ago	1 year ago	4 months ago	To hospitalization
ART adhesion	-	NO	NO	YES	-
Hospitalization time(days)	43	24	64	22	27
Tuberculosis scheme	RHZE	RHZE	RHZE	RHZE	RHZE
Reinternation	NO	NO	YES	NO	NO
Tuberculosis extra-meningeal	Pulmonary	Pulmonary	Óssea	Miliar	Ganglionar
Outcome	Discharge	Hospital transfer	Discharge	Death	Discharge

ART, antiretroviral therapy. CD4 nadir, first CD4 T lymphocyte count.

**Table 2:** CSF analysis of tuberculous meningitis cases in people with HIV/AIDS with positive AFB and/or detectable TRM-TB.

Exams	Case 1	Case 2	Case 3	Case 4	Case 5
Color	Cloudy	Light	Xanthochromic	Cloudy	Cloudy
Proteins (mg/dL)	126.7	184.1	182.4	252	237
Glucose (mg/dL)	13	54	4	52	22
Cellularity (cell/mm³)	400	125	55	250	760
Polymorphonuclear (%)	75	71	47	89	37
Mononuclear (%)	25	29	53	11	63
Acid-alcoholic resistant bacilli search	Positive	Positive	Positive	Positive	Positive
Culture of mycobacteria	M.T.^1^	M.T.^1^	NHC^2^	M.T.^1^	M.T.^1^
TRM-TB	Detected	Detected	Not detected	Detected	Detected
Molecular test	GeneXpert MTB/Rif	GeneXpert MTB/Rif	GeneXpert MTB/Rif	GeneXpert MTB/Rif	UltraXpert MTB/Rif

## Abbreviations

TB: Tuberculosis
PLWHA: People living with HIV/AIDS
AIDS: Acquired immunodeficiency syndrome
HIV: Human immunodeficiency virus
TM: Tuberculous meningitis
ART: Antiretroviral therapy
CSF: Cerebrospinal fluid
AARB: Acid-alcoholic resistant bacilli
MR: Magnetic resonance
GeneXpert MTB/RIF: *Mycobacterium tuberculosis* and detect resistance to rifampicin
TCD4: TCD4 lymphocytes.
